# A real-world study on clinical features and prognosis of Chinese breast cancer patients with brain metastases

**DOI:** 10.3389/fonc.2025.1497269

**Published:** 2026-01-07

**Authors:** Hong-dan Chen, Xiao-wen Liao, Xiao-geng Chen, Yu-nan Su, Ding-long Pan, Guo-zhong Chen, Min Wu, Shuang-long Cai

**Affiliations:** 1 First Department of Cadre Clinic, Shengli Clinical Medical College of Fujian Medical University, Fujian Provincial Hospital, Fuzhou University Affiliated Provincial Hospital, Fuzhou, Fujian, China; 2 Department of Radiation Oncology, The Second Affiliated Hospital of Fujian Medical University, Quanzhou, Fujian, China; 3 Department of Breast Surgery, Shengli Clinical Medical College of Fujian Medical University, Fujian Provincial Hospital, Fuzhou University Affiliated Provincial Hospital, Fuzhou, Fujian, China; 4 Department of Emergency, The Second Affiliated Hospital of Fujian Medical University, Quanzhou, Fujian, China; 5 Department of Thyroid and Breast Surgery, Comprehensive Breast Health Center, The Lishui Hospital of Wenzhou Medical University, The First Affiliated Hospital of Lishui University, Lishui People's Hospital, Lishui, Zhejiang, China

**Keywords:** breast cancer, brain metastases, real-world study, clinical features, prognosis

## Abstract

**Background:**

This study aimed to analyze the clinical characteristics and prognosis of breast cancer (BC) patients with brain metastases (BM).

**Methods:**

We performed a retrospective analysis of breast cancer patients with brain metastases (BCBM) in a real-world setting.

**Results:**

In a cohort of 249 breast cancer brain metastasis (BCBM) patients (all female; median age 46 years), molecular subtypes were distributed as follows: luminal (38.95%), HER2-positive (32.93%), and triple-negative (28.11%). Distinct metastatic patterns were observed: luminal subtype correlated with bone metastases (55.73%, p<0.001), HER2-positive with liver metastases (46.34%, p<0.001), and luminal with leptomeningeal metastases (19.59%, p=0.002). For CNS-directed treatment, 70.28% received radiotherapy (69.71% whole-brain radiotherapy, 30.28% stereotactic radiosurgery), while 23.69% received no local treatment. After median follow-up of 63.1 months, 81.52% had died. Multivariable analysis identified HER2-positive subtype and brain metastasis as first metastatic site as protective for overall survival after brain metastasis (OS-BM), while leptomeningeal metastasis were independent risk factors.

**Conclusion:**

This study reveals distinct patterns of metastatic spread across breast cancer molecular subtypes and identifies key prognostic factors for survival after brain metastasis. The findings underscore the critical influence of tumor biology on disease progression and outcomes, highlighting the need for subtype-specific management strategies in BCBM patients.

## 1 Introduction

Brain metastases (BM) represent one of the most significant causes of mortality in patients with malignant tumors. The incidence of brain metastasis in breast cancer patients ranks second among all malignant tumors, accounting for approximately 10% to 16% of all BM, surpassed only by lung cancer (1, 2). With recent advancements in brain imaging technology and systemic therapies, the incidence of BM is increasing. It has been reported that 5.1% of newly diagnosed breast cancer (BC) patients will develop BM within one year (3, 4), with the proportion increasing to 30% among metastatic BC patients (5). Since most antitumor drugs do not effectively cross the blood-brain barrier, patients with breast cancer brain metastases (BCBM) generally experience poor quality of life and an unfavorable prognosis (6, 7). Current data indicate that the median survival time for BCBM patients is less than six months, with a mortality rate of approximately 80% within one year (8). Furthermore, the prognosis for BCBM patients has not significantly improved over the past decades (9).

The incidence and survival rates of BM appear to vary across different BC subtypes (10, 11). Studies have shown that triple-negative breast cancer (TNBC) and HER2-positive BC patients are at a higher risk of developing BM compared to those with luminal-type BC (12, 13). TNBC exhibits the shortest median survival time when compared to luminal and HER2-positive types of BC (14). In this study, we aimed to analyze the clinical features and prognosis of BCBM patients in a real-world setting to provide valuable insights for the clinical diagnosis and treatment of BCBM.

## 2 Materials and methods

### 2.1 Patients

This retrospective analysis included 249 BCBM patients who were pathologically confirmed and treated at the Second Affiliated Hospital of Fujian Medical University from February 1, 2000, to June 1, 2023. Male patients, those with a history of other malignant tumors, or patients without BM were excluded from the study.

Clinical staging was performed according to the 7th edition of the TNM classification for breast cancer promulgated by the American Joint Committee on Cancer (AJCC). The status of estrogen receptor (ER), progesterone receptor (PR), and HER2 was determined through immunohistochemistry or *in situ* hybridization analysis of tissues from primary or metastatic lesions. Tumors with ≥1% of cells positive for ER and/or PR were classified as ‘ER and/or PR positive.’ HER2 overexpression/amplification was defined by a 3+ immunohistochemical score or a positive result in fluorescence *in situ* hybridization. Based on these immunohistochemical results, the patients were categorized into three groups: Luminal subtype group (ER and/or PR positive and HER2 negative), HER2-positive group, and triple-negative group (ER, PR, and HER2 negative).Due to inconsistent Ki-67 index data and lack of routine PR re-testing in this retrospective cohort, further subclassification into Luminal A and Luminal B subtypes was not feasible. All immunohistochemical staining and laboratory procedures were performed following strictly standardized and clinically validated protocols that were in place at our institution during the respective time periods. All staining results were interpreted by experienced two pathologists and were used for critical clinical decision-making.

### 2.2 Systemic treatment regimens

During the study period, common systemic treatment regimens included: chemotherapy (anthracycline-based, taxane-based, platinum-based agents, etc.); endocrine therapy (aromatase inhibitors, tamoxifen, fulvestrant [for patients with luminal disease]); and targeted therapy (trastuzumab, pertuzumab [for HER2-positive patients]). A proportion of patients with more advanced disease received immunotherapy (e.g., immune checkpoint inhibitors). Specific treatment plans were selected based on tumour subtype, prior therapy, clinical performance status, and patient preference.

### 2.3 BM diagnosis and follow-up

The diagnosis of brain metastases (BM) is primarily based on clinical manifestations and cranial imaging examinations. Clinical symptoms may include unexplained headaches, vomiting, sensory or motor peripheral/central nervous system symptoms, as well as abnormalities in defecation and urination. Cranial imaging examinations typically involve both plain and enhanced CT scans and MRI scans. Routine practice employs cranial plain and enhanced MRI scans, while plain and enhanced CT scans are recommended for patients with contraindications to MRI. In some cases, where surgery was performed to remove brain metastases, the diagnosis of BM was confirmed through pathological examination.

All breast cancer (BC) patients included in our study were monitored primarily via telephone follow-up or visits to our outpatient clinic, with results recorded meticulously. The follow-up period commenced from the initial diagnosis of BC and continued until the most recent follow-up date, which was gathered up to June 1, 2023. The time from the diagnosis of BC to the diagnosis of BM (TTBM) was defined as the duration from the first diagnosis of breast cancer to the identification of brain metastases. The time from the first recurrence to BM diagnosis (TFR-BM) was defined as the interval from the detection of the first recurrence to the discovery of brain metastases. The first recurrence encompassed both local and distant recurrences of BC. The overall survival from BM diagnosis (OS-BM) was defined as the period from the identification of brain metastases to death or the last follow-up. Finally, the overall survival from BC diagnosis (OS) was determined as the duration from the first diagnosis of breast cancer to death or the last follow-up.

### 2.4 Treatments for BM

The treatments for BCBM patients were guided by major clinical guidelines and recommendations. Local treatment was prioritized as the preferred modality, while systemic treatment was considered a supplementary approach. Additionally, specific patient and family factors such as economic conditions and patient preferences were taken into account. For symptomatic BCBM patients requiring urgent local brain treatment or those with leptomeningeal metastases, local treatment (either radiotherapy or surgery for BM) was the preferred option. Conversely, asymptomatic BCBM patients were directed towards systemic treatment as the first line of management. During treatment, the effectiveness of the interventions was regularly evaluated, with local treatment implemented in cases of intracranial lesion progression.

### 2.5 Statistical analysis

Statistical analyses were performed using IBM SPSS Statistics, Version 22.0 (Armonk, NY: IBM Corp). Univariate analysis utilized a one-way ANOVA test, chi-square test, and Fisher’s exact test. A one-way ANOVA test was conducted for the comparison of quantitative indicators among the three groups, while the chi-square test and Fisher’s exact test were employed for comparing sample rates across these groups. The Kaplan-Meier method was used for survival analysis of all patients, and the log-rank test was applied to analyze prognostic differences among groups. P-values <0.05 were considered statistically significant.

## 3 Results

A total of 249 patients with breast cancer brain metastases (BCBM) were identified, all of whom were female, with a median age of 46 years (range: 23–75 years). Among these patients, 97 (38.95%) had luminal subtype breast cancer, 82 (32.93%) had HER2-positive subtype breast cancer, and 70 (28.11%) had triple-negative breast cancer (TNBC). The most common initial distant metastatic sites in the study population included the lung (151 cases, 60.64%), bone (124 cases, 49.80%), liver (67 cases, 26.91%), and brain (68 cases, 27.31%). Notably, luminal subtype patients exhibited a higher proportion of bone metastases (55.73%, P < 0.001), while HER2-positive patients accounted for the majority of liver metastases (46.34%, P < 0.001). Although TNBC patients had the highest proportion of BM (32.86%), this difference was not statistically significant compared to the other two subtypes. Throughout the disease course, luminal subtype patients were more likely to develop leptomeningeal metastases (19.59%, P = 0.002 < 0.05) (see **Table 1**).

**Table 1:** Clinical characteristics of 249 breast cancer patients with brain metastases.

Variables	Total (n=249)	Molecular subtypes			P value
		LUMINAL (n=97)	HER2 POSITIVE (n=82)	TNBC (n=70)	
	No.(%)	No.(%)	No.(%)	No.(%)	
Age (years)	46	46	46.5	47.5	0.674
Median(range)	(23, 75)	(23, 66)	(23, 70)	(27, 75)	
Menopausal status at diagnosis					0.875
Premenopausal	166 (66.67%)	66 (68.04%)	55 (67.07%)	45 (64.29%)	
Postmenopausal	83 (33.33%)	31 (31.96%)	27 (32.93%)	25 (35.71%)	
Family history					0.944
No	223 (89.56%)	87 (89.69%)	74 (90.24%)	62 (88.57%)	
Yes	26 (10.44%)	10 (10.31%)	8 (9.76%)	8 (11.43%)	
Pathology type					0.561
IDC	212 (85.14%)	84 (86.60%)	69 (84.15%)	59 (84.29%)	
IBC	5 (2.01%)	2 (2.06%)	0 (0.00%)	3 (4.29%)	
Mix	26 (10.44%)	8 (8.25%)	11 (13.41%)	7 (10.00%)	
Others	6 (2.41%)	3 (3.09%)	2 (2.44%)	1 (1.43%)	
TNM staging					0.006
0	3 (1.20%)	0 (0.00%)	0 (0.00%)	3 (4.29%)	
I	38 (15.26%)	18 (18.56%)	10 (12.20%)	10 (14.29%)	
II	73 (29.32%)	28 (28.87%)	16 (19.51%)	29 (41.43%)	
III	82 (32.93%)	31 (31.96%)	29 (35.37%)	22 (31.43%)	
IV	39 (15.66%)	15 (15.46%)	19 (23.17%)	5 (7.14%)	
Missing	14 (5.62%)	5 (5.15%)	8 (9.76%)	1 (1.43%)	
First metastatic site					
Lung metastases					0.337
No	98 (39.36%)	42 (43.30%)	27 (32.93%)	29 (41.43%)	
Yes	151 (60.64%)	55 (56.70%)	55 (67.07%)	41 (58.57%)	
Liver metastases					<0.001
No	182 (73.09%)	77 (79.38%)	44 (53.66%)	61 (87.14%)	
Yes	67 (26.91%)	20 (20.62%)	38 (46.34%)	9 (12.86%)	
Bone metastases					<0.001
No	125 (50.20%)	34 (35.05%)	37 (45.12%)	54 (77.14%)	
Yes	124 (49.80%)	63 (64.95%)	45 (54.88%)	16 (22.86%)	
Brain metastases					0.224
No	181 (72.69%)	69 (71.13%)	65 (79.27%)	47 (67.14%)	
Yes	68 (27.31%)	28 (28.87%)	17 (20.73%)	23 (32.86%)	
Metastatic site in the chest wall or regional lymph nodes					0.5
No	83 (33.33%)	29 (29.90%)	27 (32.93%)	27 (38.57%)	
Yes	166 (66.67%)	68 (70.10%)	55 (67.07%)	43 (61.43%)	
Number of brain metastasis					0.693
Single	84 (34.57%)	31 (32.98%)	31 (32.98%)	26 (38.81%)	
Multiple	159 (65.43%)	63 (67.02%)	55 (67.07%)	41 (61.19%)	
Leptomeningeal metastases					0.002
No	220 (88.35%)	78 (80.41%)	80 (97.56%)	62 (88.57%)	
Yes	29 (11.65%)	19 (19.59%)	2 (2.44%)	8 (11.43%)	
Local treatments for brain metastases					0.084
RT only	149 (59.84%)	65 (67.01%)	48 (58.54%)	36 (51.43%)	
Surgery alone	15 (6.02%)	2 (2.06%)	9 (10.98%)	4 (5.71%)	
Surgery+RT	26 (10.44%)	8 (8.25%)	10 (12.20%)	8 (11.43%)	
No Local treatment	59 (23.69%)	22 (22.68%)	15 (18.29%)	22 (31.43%)	

TNBC, Triple-Negative Breast Cancer; RT, Radiotherapy; HER2, Human Epidermal Growth Factor Receptor 2; IDC, Invasive Ductal Carcinoma; IBC, Invasive Lobular Carcinoma.

Family history: Hereditary Breast and Ovarian Cancer (HBOC) Related Cancer History

In terms of CNS-directed treatment, 175 patients (70.28%) underwent radiotherapy for brain metastases. Among these, most patients (69.71%) received whole-brain radiotherapy, while a minority (30.28%) underwent stereotactic radiosurgery. Additionally, 41 patients (16.46%) required neurosurgery, and 26 patients (10.44%) received both radiotherapy and neurosurgery for their brain lesions. Conversely, 59 patients (23.69%) did not receive any local treatments for brain metastases. Systemic disease control rates following BM diagnosis varied by subtype and treatment line, but overall, intracranial disease control was challenging, with many patients experiencing progression despite multimodal therapy.

The median follow-up time for the cohort was 63.1 months (95% CI: 54.6-72.4 months). At the most recent follow-up, 203 patients (81.52%) had died, while 46 (18.47%) were still alive. Over the entire disease course, 84 patients (34.57%) had single BM, whereas 159 patients (65.43%) had multiple BM; the number of BM in 6 patients was not documented. Additionally, 29 patients (11.65%) experienced leptomeningeal metastases. Among the 82 patients with HER2-positive breast cancer, 11 did not receive anti-HER2-targeted therapy after BM diagnosis, while 68 patients did receive such treatment; information on 3 additional patients regarding subsequent anti-HER2-targeted therapy was missing.

**Figure 1:**
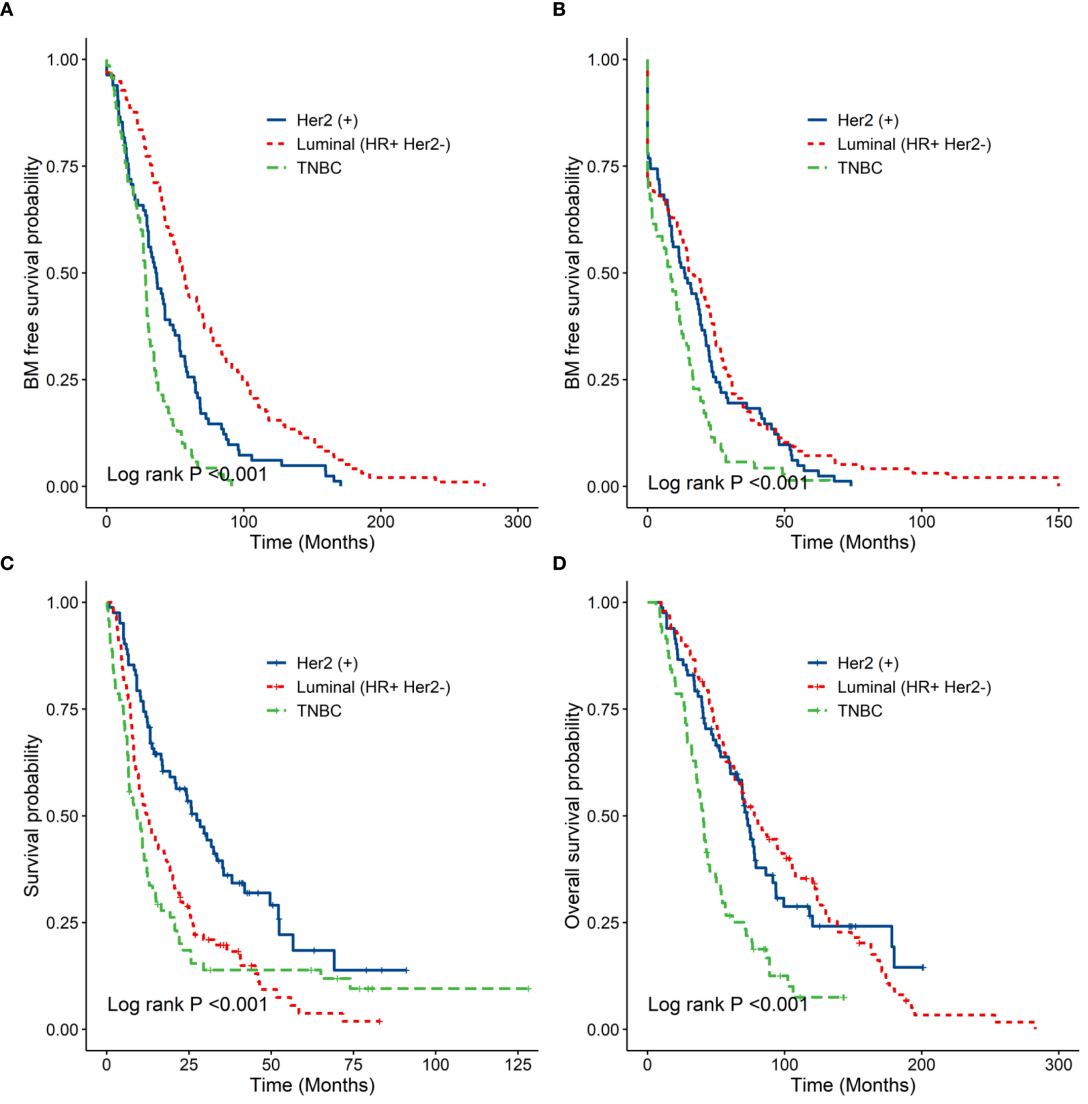
**(A)** showed the time from diagnosis of BC to diagnosis of BM. **(B)** showed the time from first recurrence to diagnosis of BM. **(C)** showed from diagnosis of BM to death or follow-up time. **(D)** showed the time from diagnosis of BC to death or follow-up time.

**Figure 2:**
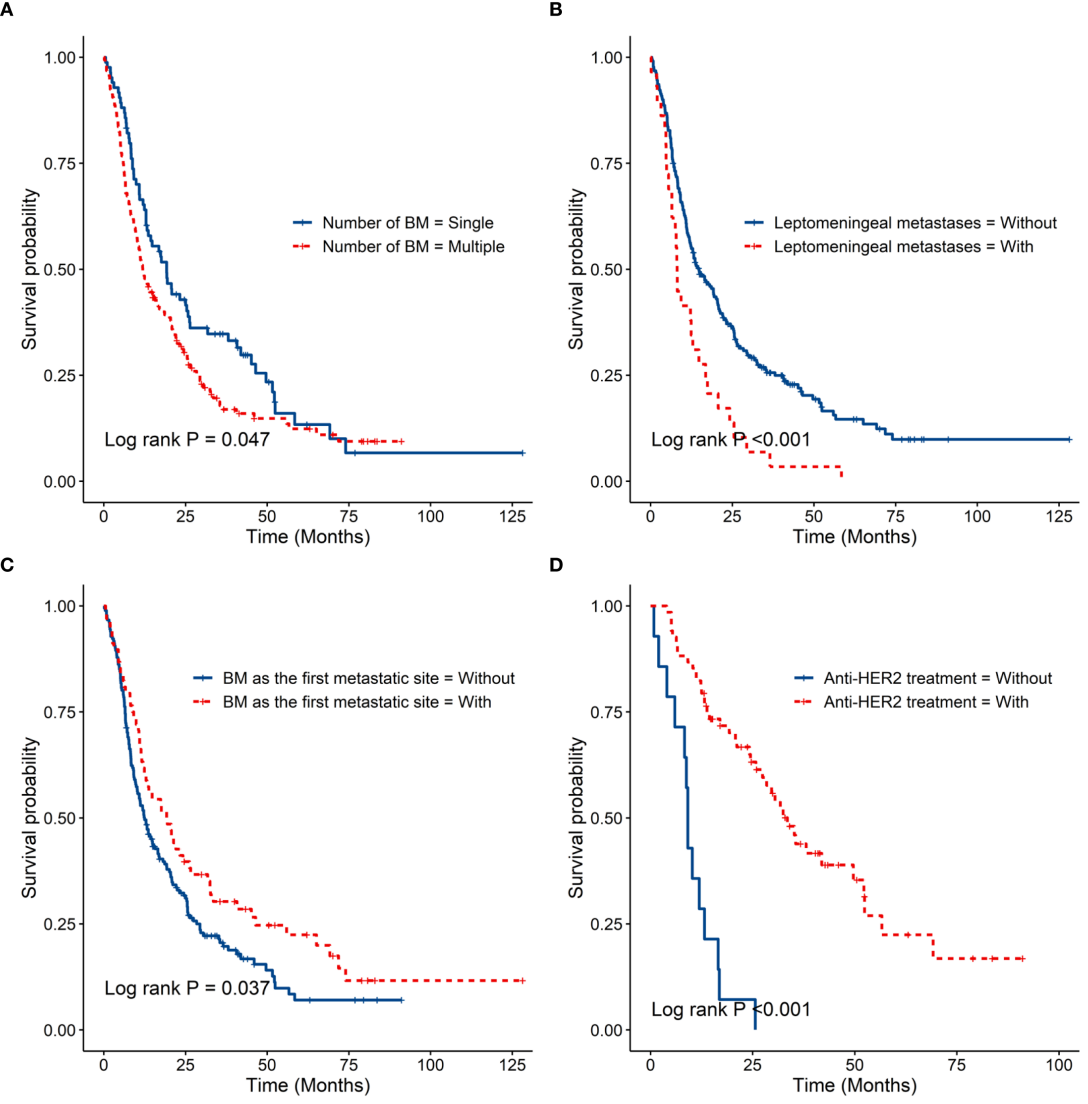
**(A)** showed the overall survival from BM diagnosis with different numbers of BM. **(B)** showed the overall survival from BM diagnosis with or without leptomeningeal metastases. **(C)** showed the overall survival from BM diagnosis with or without BM as the first metastatic site. **(D)** showed the overall survival from BM diagnosis with or without Anti-HER2 treatment for Her2 positive breast cancer patients.

The median time from diagnosis of BC to BM diagnosis (TTBM) was 56.6 months for luminal BC, 36.1 months for HER2+ BC, and 28.2 months for TNBC (P < 0.001). The median overall survival after BM diagnosis (OS-BM) was 12.2 months for luminal BC, 27.4 months for HER2+ BC, and 9.2 months for TNBC (P < 0.001) (**Table 2**, **Figures 1A–D**). The OS-BM between patients with single versus multiple BM was statistically different (19.2 vs. 11.9 months, P < 0.05). There was a significant difference in OS-BM between patients with and without LM (8.1 vs. 14.8 months, P < 0.001). Additionally, OS-BM differed significantly between patients with BM as the first metastatic site and those without (19.3 vs. 12.5 months, P < 0.05). Furthermore, the difference in OS-BM between HER2+ patients receiving anti-HER2-targeted therapy and those not treated was significant (33.5 vs. 9.1 months, P < 0.001). (**Table 3**, **Figures 2A–D**). To account for the evolution of anti-HER2 therapy, we stratified HER2+ patients into pre-2013 (n=28) and post-2013 (n=54) eras. The post-2013 group showed a significantly longer OS-BM (38.1 vs. 11.1 months, P<0.001), likely reflecting improved treatment options.

**Table 2:** Median time for breast cancer patients with brain metastases in different clinical progressions.

Clinical progressions		Median time(months)				P value
From	To	All patients (N=249)	Molecular subtypes			
			LUMINAL (n=97)	HER2 POSITIVE (n=82)	TNBC (n=70)	
BC	BM	37 (32.9-42.5)	56.6 (46.4-70.3)	36.1 (30.5-46.1)	28.2 (25.1-30.8)	<0.001
FR	BM	12.2 (9.33-15.3)	16.67 (11.90-23.5)	13.57 (8.80-19.3)	8.42 (3.07-12.5)	<0.001
BM	DOFU	13.6 (11.6-18.1)	12.2 (9.63-18.6)	27.4 (19.23-35.5)	9.2 (6.63-12.2)	<0.001
BC	DOFU	63.1 (54.6-72.4)	77.9 (63.7-105.5)	72.4 (60.5-86.3)	40.5 (35.7-49.7)	<0.001

TNBC, Triple-Negative Breast Cancer; HER2, Human Epidermal Growth Factor Receptor 2.

From BC To BM: The median time from the first diagnosis of breast cancer to the identification of brain metastases; From FR To BM: The median time from the detection of the first recurrence to the discovery of brain metastases; From BM To DOFU: The median time from BM diagnosis to death or follow-up; From BC To DOFU: The median time from the first diagnosis of breast cancer to death or follow-up;

CI, Confidence Interval Data are presented as median(months)(95%CI).

**Table 3:** Analysis of the median time from diagnosis of brain metastases to death or follow-up in different BCBM patients.

Stratified variables		Median time(month)	P value
Number of BM	Single (n=84)	19.2 (13.2-26.2)	0.047
	Multiple (n=159)	11.9 (10.2-16.6)	
Leptomeningeal metastases	With (n=29)	8.13 (6.53-14.8)	<0.001
	Without (n=220)	14.80 (12.23-20.4)	
BM as the first metastatic site	With (n=68)	19.3 (12.4-31.8)	0.037
	Without (n=181)	12.5 (10.2-16.6)	
Anti-HER2 treatment	With (n=68)	33.50 (27.40-52.2)	<0.001
	Without (n=11)	9.13 (8.33-16.8)	

BM, Brain Metastases; BCBM, Breast Cancer Brain Metastases; HER2, Human Epidermal Growth Factor Receptor 2; CI, Confidence Interval.

Analysis Methodology: Median survival was analyzed using Kaplan-Meier analysis. Data are presented as median months with corresponding 95% confidence intervals.

COX univariable and multivariable analyses identified the following as independent factors influencing overall survival after brain metastasis (OS-BM): the HER2-positive molecular subtype of breast cancer (protective factor), brain metastasis as the first site of metastasis (protective factor), and leptomeningeal metastasis (risk factor) (**Table 4**).

**Table 4:** COX univariate and multivariate analysis of overall survival from BM diagnosis.

Variables	Univariate analyses			Multivariate analyses		
	HR	95%CI	P	HR	95%CI	P
Age						
≤40 years	1			—	—	—
>40 years	1.103	0.797-1.526	0.555	—	—	—
Menopausal status at diagnosis						
Premenopausal	1			—	—	—
Postmenopausal	1.15	0.859-1.541	0.347	—	—	—
Family history						
No	1			—	—	—
Yes	0.657	0.403-1.069	0.091	—	—	—
Molecular Subtype						
HR Positive Her2 Negative	1			1		
TNBC	1.141	0.819-1.589	0.437	1.261	0.901-1.765	0.176
Her2 Positive	0.544	0.388-0.764	<0.001	0.555	0.39-0.789	0.001
Lung Metastasis as First Metastatic Site						
No	1			—	—	—
Yes	1.059	0.799-1.403	0.692	—	—	—
Liver Metastasis as First Metastatic Site						
No	1			—	—	—
Yes	1.093	0.803-1.487	0.573	—	—	—
Bone Metastasis as First Metastatic Site						
No	1			—	—	—
Yes	1.033	0.784-1.361	0.819	—	—	—
Brain Metastasis as First Metastatic Site						
No	1			1		
Yes	0.717	0.524-0.981	0.038	0.649	0.47-0.897	0.009
Chest Wall and Regional Lymph Nodes Metastasis as First Metastatic Site						
No	1			—	—	—
Yes	1.308	0.969-1.767	0.079	—	—	—
Numbers of Brain Metastases						
Single	1			1		
Multiple	1.384	1.029-1.861	0.032	1.347	1-1.815	0.0501
Leptomeningeal Metastases						
No	1			1		
Yes	1.961	1.319-2.916	0.001	1.653	1.098-2.488	0.016

## 4 Discussion

With advancements in imaging technology and the enhancement of comprehensive treatment options, the survival time for breast cancer (BC) patients has significantly increased in recent years. As a result, BC patients with prolonged survival may exhibit a greater tendency to develop brain metastases (BM). Consequently, the incidence of BCBM appears to be rising. Our real-world analysis of 249 patients with breast cancer brain metastases (BCBM) elucidates the profound heterogeneity in metastatic patterns, treatment approaches, and survival outcomes across molecular subtypes, providing contemporary evidence to refine the management of this challenging condition.

Our findings firmly establish the molecular subtype as a cornerstone of the BCBM disease trajectory. Consistent with prior literature (15–17), patients with triple-negative breast cancer (TNBC) experienced the shortest median time from breast cancer diagnosis to BM (TTBM) and the poorest overall survival after BM (OS-BM), underscoring the aggressive nature and limited therapeutic options for this subtype. In contrast, patients with HER2-positive disease, while exhibiting a high risk and short TTBM, demonstrated the most favorable OS-BM, with a median survival of 27.4 months. This “survival paradox” is largely attributable to the widespread use and intracranial efficacy of anti-HER2 targeted therapies (18, 19). Our data provide robust support for this, showing a dramatic extension in OS-BM for HER2-positive patients who received anti-HER2 therapy compared to those who did not (33.5 months vs. 9.1 months). This underscores the foundational importance of continuing effective systemic therapy beyond the diagnosis of BM in the modern treatment paradigm for HER2-positive disease (20, 21).

We observed distinct patterns of organotropism among the subtypes. Luminal patients had a higher propensity for bone metastases, whereas HER2-positive patients accounted for the majority of liver metastases, aligning with the intrinsic biological behavior of these cancers (22, 23). A notable finding was the significantly higher incidence of leptomeningeal metastases (LM) in the luminal subtype. Although luminal breast cancer is often characterized by a more indolent course, its specific tropism for the leptomeninges suggests unique tumour-microenvironment interactions and blood-brain barrier penetration mechanisms that warrant further translational investigation (24, 25).

Multivariable Cox regression analysis identified three independent prognostic factors for OS-BM: HER2-positive subtype (protective factor), BM as the first site of distant metastasis (protective factor), and the development of LM (risk factor). BM as the first metastatic site likely portends a better prognosis because it is associated with a lower systemic tumour burden and better performance status, allowing for more aggressive and timely local and systemic interventions (26). Conversely, LM represents a catastrophic prognostic indicator, with our data confirming a median OS-BM of only 8.1 months, highlighting the critical need for improved early detection and novel therapeutic strategies for this complication (27, 28).

Our real-world treatment data reflect contemporary clinical practice. Whole-brain radiotherapy remained the most common local therapy, although stereotactic radiosurgery was used in a substantial minority of cases, reflecting an effort to preserve neurocognitive function in selected patients (29, 30). However, nearly a quarter of the cohort received no local therapy for their BM, a decision likely influenced by poor performance status, extensive extracranial disease, or patient preference, pointing to an area where palliative and supportive care can be optimized (31–33).

## 5 Limitations

Our study has several limitations. Its retrospective, single-institute design introduces potential for selection bias. The 23-year study period encompasses evolving standards for diagnosis, staging, and treatment, particularly for HER2+ disease, creating patient heterogeneity. Molecular subtyping was based primarily on the primary tumor IHC/FISH, and re-biopsy of metastatic lesions was not routine; thus, subtype conversion could not be assessed, and further biomarker validation (e.g., Ki-67, PIK3CA, BRCA) was not feasible for most patients, limiting deeper biological insights. The sample size, while substantial for a single-center BCBM study, remains relatively modest for extensive subgroup analyses. Details on some systemic treatment regimens and systemic disease control metrics were incomplete in some historical records. Future prospective, multi-center studies with larger cohorts and comprehensive biomarker profiling are warranted.

## 6 Conclusion

In summary, our real-world analysis elucidates subtype-specific patterns of metastasis and identifies key prognostic factors in BCBM patients. These insights reinforce the importance of molecular subtyping in guiding surveillance and treatment strategies. As therapeutic options continue to expand, individualized approaches that integrate tumor biology, metastatic burden, and patient performance status will be essential to improving outcomes in this high-risk population.

## Data Availability

The original contributions presented in the study are included in the article/supplementary material. Further inquiries can be directed to the corresponding authors.
